# Confirmatory and bi-factor analysis of the Short Form Health Survey 8 (SF-8) scale structure in a German general population sample

**DOI:** 10.1186/s12955-021-01699-8

**Published:** 2021-03-03

**Authors:** M. A. Wirtz, A. Schulz, E. Brähler

**Affiliations:** 1 Research Methods, Institute of Everyday Culture, Sports and Health, University of Education Freiburg, Kunzenweg 21, 79117 Freiburg, Germany; 2 Department of Medical Psychology and Medical Sociology, University of Leipzig, Leipzig, Germany; 3 Clinic for Psychosomatic Medicine and Psychotherapy, University Medical Center of the Johannes Gutenberg University Mainz, Mainz, Germany

**Keywords:** Short form Health Survey 8 (SF-8), Health-related quality of life (HRQoL), Construct validity, Confirmatory structural modelling, Bi-factor model

## Abstract

**Background:**

The SF-8 is a short form of the SF-36 Health Survey, which is used for generic assessment of physical and mental aspects of health-related quality of life (HRQoL). Each of the 8 dimensions of the SF-36 is covered by a single item in the SF-8. The aim of the study was to examine the latent model structure of the SF-8.

**Method:**

One-, two- and three dimensional as well as bi-factor structural models were defined and estimated adopting the ML- as well as the WLSMV-algorithm for ordinal data. The data were collected in a German general population sample (N = 2545 persons).

**Results:**

A two- (physical and mental health) and a three-dimensional CFA structure (in addition overall health) represent the empirical data information adequately [CFI = .987/.995; SRMR = .024/.014]. If a general factor is added, the resulting bi-factor models provide a further improvement in data fit [CFI = .999/.998; SRMR = .001]. The individual items are much more highly associated with the general HRQoL factor (loadings: .698 to .908) than with the factors physical, mental, and overall health (loadings: −.206 to .566).

**Conclusions:**

In the SF-8, each item reflects mainly general HRQoL (general factor) as well as one of the three components physical, mental, and overall health. The findings suggest in particular that the evaluation of the information of the SF-8 items can be validly supplemented by a general value HRQoL.

**Supplementary Information:**

The online version contains supplementary material available at 10.1186/s12955-021-01699-8.

## Introduction

A comprehensive understanding of health requires considering the health status of people based on a bio-psycho-social model [1]. Accordingly, the construct of health-related quality of life (HRQoL) has been established as the third central outcome parameter in health research—in addition to mortality and morbidity. HRQoL is understood as a multidimensional construct. HRQoL reflects subjectively reported aspects of physical and mental health of individuals and the impact of the health status on QoL [2–4].

The Short-Form-36 (SF-36 [5–7]) is one of the most frequently used instruments for HRQoL assessment in international health research. With 36 items, the instrument records aspects of physical, mental and social health from the subjective perspective of the respondents. Based on the answers to the 36 single items, the values on the 8 underlying single constructs *Physical Functioning (PF)*, *Physical Role Functioning (PR)*, *Bodily Pain (BP)*, *General Health (GH)*, *Vitality (VT)*, *Social Functioning (SF)*, *Emotional Role Functioning (RE)* and *Mental Health (MH)* can be determined. Additionally, the values on these 8 dimensions can be aggregated to a *Physical Component Summary (PCS)* value and a *Mental Component Summary (MCS)*.

### *Original factorial SF-8 structure proposed by Ware *et al*. *[8]

**Fig. 1:**
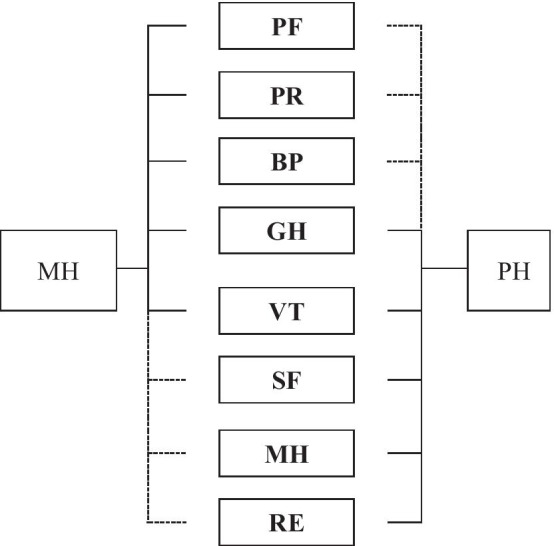
Structure of the full and restricted (without loadings marked with dashed lines) WIM-models according to Ware et al. [8]. *PF* Physical Functioning, *PR* Physical Role Functioning, *BP* Bodily Pain, *GH* General Health, *VT* Vitality, *SF* Social Functioning, *RE* Role Functioning Emotional, *PH* Physical Health, *MH* Mental Health

To provide a time-efficient screening of physical and mental aspects of HRQoL the SF-8 has been developed. In the SF-8 each of the 8 SF-36 dimensions is represented by a single item [6]. In their original study Ware et al. [8] applied a principal component analysis (PCA) to identify the factorial structure of the SF-8 (see Fig. 1; full model). Factor loadings were allowed for all 8 single items on each of the two uncorrelated constructs PCS and MCS. Nevertheless, both constructs proved to be mainly represented by 6 items. The physical component *PCS* reflects *Physical Functioning*, *Physical Role Functioning*, *Bodily Pain, General Health and Vitality*. The *mental* component *MCS* mainly represents the facets *Social Functioning*, *Mental Health, Emotional Role Functioning, General Health and Vitality* [8]. Accordingly, *General Health and Vitality* proved to be germane indicators of both underlying constructs *PCS and MCS* (see Fig. 1; restricted model structure).

### Confirmatory factorial analyzes of the SF-8 structure

**Fig. 2:**
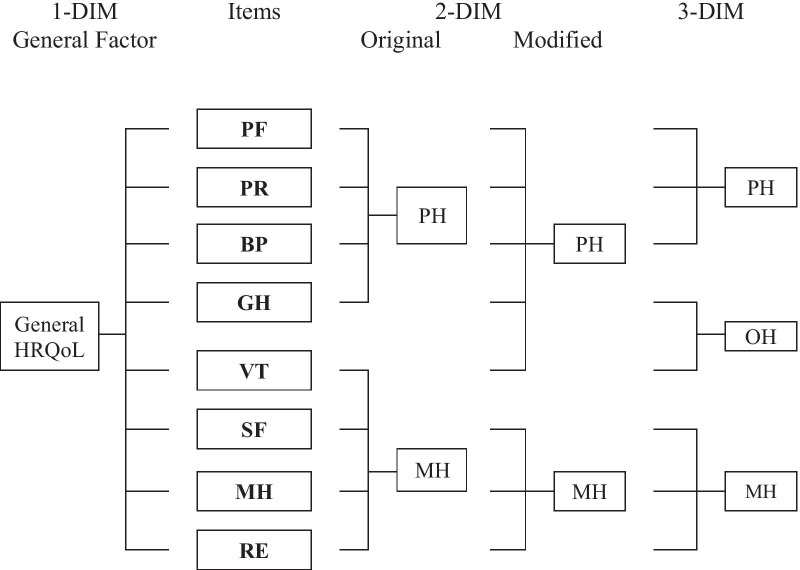
Factorial model structures of the SF-8 according to Lang et al. [10]. *PF* Physical Functioning, *PR* Physical Role Functioning, *BP* Bodily Pain, *GH* General Health, *VT* Vitality, *SF* Social Functioning, *MH* Mental Health, *RE* Role Functioning Emotional, *PH* Physical Health, *OH* Overall Health

Wang et al. [9] as well as Lang et al. [10] used a confirmatory factor analytical (CFA) approach to investigate the underlying latent structure of the SF-8. In CFA models, a theory-based specification is made for each item to which latent variable it is assigned. CFA models assuming between-item-multidimensionality (BIM) require that each item loads on only one factor. Wang et al. [9] as well as Lang et al. [10] identified a three dimensional BIM structure as the best fitting model in Chinese samples. The third factor *Overall Health* is reflected by the item pair *General Health* and *Vitality* (see Fig. 2; 3-DIM). Lang et al. [10] emphasize that this result for the SF-8 is consistent with studies on the SF-36, which have shown a third component of *General Well-Being* besides *Physical* and *Mental Health* to be relevant [11–15].

Furthermore, Lang et al. [10] found a two-dimensional CFA model to be acceptable (see Fig. 2; 2-DIM). Nevertheless, the item *Vitality* showed a noticeably weak item-total correlation. On closer examination of the data reported by Lang et al. [10], this seems quite reasonable: the item *Vitality* is closely related to the item *General Health*, which is clearly assigned to *Physical Health* in the two-factor model. Accordingly, *Vitality* should be considered as an indicator of *Physical Health* rather than *Mental Health*. This model structure corresponds exactly to the structure that Hann and Reeves [16] found valid for the SF-36. In Fig. 2 the 2-DIM-Modified model represents the corresponding latent model structure.

For the SF-36 [16, 17], the SF-12 [18, 19] and the SF-8 [9, 10] the underlying constructs proved to be highly correlated. Nevertheless, the assumption that a general component is reflected in all SF-items could not be confirmed (unidimensional model; see Fig. 2: 1-DIM), because multifactorial models provided a better data fit.

### Bi-factor models of the SF-8 structure

Bi-factor models consider the answer to each item to be determined by two information components simultaneously (with2in-item-multidimensionality; WIM; [20, 21]). Regarding the construct HRQoL, each item has to be assigned to a general (i.e. general HRQol) and a specific latent variable (i.e. physical, mental or overall). As shown in Fig. 2, three bi-factor models can be defined for the SF-8 by combining the single factor model (1-DIM; left) with one of the three multi-dimensional models (2-DIM, 2-DIM^MOD^, 3-DIM; right). Accordingly, the response to each item reflects the general HRQoL on the one hand and an physical, mental or overall aspect on the other [22–24]. Chen, West and Sousa [25] pointed out, that bi-factor models generally provide a reasonable alternative model approach, if highly related domains comprise the general multifaceted construct of interest. The assumption that the general characteristic HRQoL value is included in the answers to each item of an HRQoL scale is in concordance with the underlying theoretical assumptions regarding HRQoL [2].

Knowing the underlying model structure is a prerequisite to validly interpret and use the information of the SF-8 items for diagnostic and evaluative purposes. Hence, the central aim of the present study was to comparatively evaluate the factor structures underlying the SF-8 items. The specific aims were:

1. To determine the fit of existing SF-8 models for a German general population sample.
  CFA models assuming both WIM (see. Fig. 1) and BIM (see Fig. 2: 2-DIM, 2-DIM^MOD^ and 3-DIM) have been evaluated.
2. To determine the fit of bi-factor models which assume a general factor HRQoL as an additional source of information.
  The three WIM models combine each of model structures in Fig. 2 on the right (2-DIM, 2-DIM^MOD^ and 3-DIM) with the general 1-DIM model (Fig. 2 on the left).

## Methods

### Data collection

The SF-8 data were collected in a multi-topic survey commissioned by the University of Leipzig and conducted by the research institute USUMA Berlin in autumn 2004. The aim of the survey was to obtain a representative sample of people living in private households in Germany aged 14 and over. In order to ensure the representativeness of the sample for the German population, a random selection of households was first made using the random route method [26]. The person to be interviewed was selected randomly in the household. The utilization rate of the survey is 62.3%. A total of N = 2591 persons between the ages of 14 and 99 were interviewed on the basis of voluntary participation.

The research institute USUMA provided weighting factors (γ_i_) for each participant. These weighting factors (γ_i_) can be used to correct violations of representativeness with regard to central socio-demographic characteristics (i.e. state, gender and age). The weights correct the increased selection probability of individuals in small households and the distortions due to the lack of participation of randomly selected individuals. Members of groups that are underrepresented (vs. overrepresented) in the sample receive a weight greater (vs. smaller) than 1, ensuring that the corrected actual values correspond to the target values in the population. These corrective weighting factors were used to determine the univariate distributions and correlation statistics.

### Statistical analysis

The SF-8 models were estimated using CFA (BIM and WIM). The CFA determines the model parameters ensuring the best fit of (a) the model-based and (b) the empirical item associations (variance–covariance matrix). The χ^2^-value allows to determine the significance of the differences between the empirical and the model-based information. However, the validity of this test is considerably limited due to its overly high testing power in large samples. Alternative measures focus on the empirical relevance of the differences [27]: According to the root mean square error of approximation, a model is considered as good fitting if less than 5% (RMSEA < 0.05) of the information in the empirical variance–covariance matrix remains unexplained (acceptable model fit: RMSEA < 0.08). The incremental fit measures Confirmatory Fit Index (CFI) and Tucker–Lewis-Index (TLI) exhibit higher values, the more information a model can explain compared to a baseline model that assumes uncorrelated items (good model fit: CFI, TLI > 0.97; acceptable model fit: CFI, TLI > 0.95, [27]).

The maximum likelihood (ML) approach assumes normally distributed data and allows the most comprehensive determination of model fit criteria [27, 28]. This procedure proves to be robust to moderate violations of the normal distribution [29]. The ML approach is generally used for the analysis of SF-8 in the literature [9, 10]. However, in the present norm data, distribution problems caused by considerable ceiling and floor effects prevail (see Table 2). The WLSMV algorithm (Weighted-Least-Squared-Means-Variance) requires only ordinally scaled data. It has proven advantageous over alternative distribution-free estimation methods (e.g. MLR; robust ML estimation) for sufficiently large samples (N > 1000) [29–31]. The statistical model assumes that the categorically measured data are based on a multivariate latent normal distribution. This is a generally plausible assumption for ordinal collected questionnaire data [32–34]. When using the WLSMV algorithm, the Standardized Root Mean Square Residual (SRMR; good fit; < 0.05) proved to be a more valid fit indicator than RMSEA, especially in large samples [35]. Despite the superiority of the WLSMV algorithm for the present data set, the findings for the ML estimates are also reported to ensure comparability with existing analyses. For all model estimates, the item loadings are freely estimated in case of more than two indicators (tau-equivalent modelling). The model estimates are performed using the software Mplus 8.0 [36].

In addition to global quality criteria, it must also be ensured at the local item level that each item is sufficiently closely associated with the factor to which it is assigned: factor loadings > 0.63 or indicator reliabilities > 0.4 indicate a sufficiently clear item-construct assignment [28].

## Results

### Sociodemograhic characteristics

Sociodemographic characteristics of the N = 2545 people in the sample are depicted in Table 1. The weighting factors γ_i_ indicate that distortions could not be avoided despite the elaborate procedure for ensuring representativeness. The last column shows the correlation between γ and the socio-demographic characteristics. The significant correlations were due to the fact that women, people in the higher age groups, people with lower income and lower education, workers and people living alone are overrepresented in the study (γ_i_ < 1). The SF-8 items were positively correlated with γ_i_ (see Table 2; column r_iγ_): People with lower HRQoL were overrepresented in the study sample.

**Table 1:** Sociodemographic characteristics of the study sample

		Male N(%);γ^1^	Female N(%);γ	Total N(%);γ	Correlation^3^ γ
		1236 (47.3%); 1.03	1309 (52.7%); 0.97	2545	r = − .058**
Age [14; 99]	M	47.5	47.7	47.6	r = − .006
	SD	17.8	18.2	18.0	
Age groups	[14–25[	150 (12.5%); 1.34	149 (11.1%); 1.22	299 (11.7%); 1.28	η_Alter_ = .270*** η_Alter × Geschlecht_ = .187***
	[25–35]	176 (14.6%); 1.07	208 (15.5%); 0.85	348 (15.1%); 0.95	
	[35–45]	232 (19.3%); 1.09	285 (21.3%); 0.94	517 (20.3%); 1.01	
	[45–55]	185 (15.4%); 1.13	213 (15.9%); 0.98	398 (15.6%); 1.04	
	[55–65]	203 (16.9%); 0.89	196 (14.6%); 1.04	399 (15.7%); 0.94	
	[65–75]	192 (15.9%); 0.80	175 (13.0%); 0.87	367 (14.4%); 0.91	
	[75–99]	66 (5.5%); 0.78	115 (8.6%); 0.98	481 (7.1%); 0.83	
School graduation	I. Secondary school certificate			1178 (46.3%); 0.98	η = .187***
	II. Middle school certificate			929 (33.7%); 0.99	
	III. High school graduation			370 (21.4%); 1.00	
	IV. Student			68 (2.7%); 1.49	
Monthly income (EUR)	I. –750]			109 (4.3%); 0.54	ρ = .512***
	II. ]750–1250]			526 (20.7%); 0.71	
	III. ]1250–2000]			943 (37.1%); 0.96	
	IV. ]2000–			856 (35.2%); 1.26	
	no information			111 (4.4%); 1.30	
Marital status	I. Married—living together			1329 (52.2%); 1.15	η = 0.432***
	II. Married—living separately			29 (1.1%); 0.80	
	III. Single			625 (24.6%); 1.00	
	III. Divorced			261 (10.3%); 0.67	
	IV. Widowed			301 (11.8); 0.66	
Most recent occupation	I. Never employed before			47 (1.8%); 1.07	η = .071^n.s^
	II. Worker			254 (10.0%); 0.97	
	III. Skilled worker			644 (25.3%); 0.95	
	IV. Independent/self-employed worker			146 (5.8%); 1.15	
	V. Employees			1140 (44.8%); 1.01	
	VI. Officer			106 (4.2%); 1.03	
	VII. no information			208 (8.2%); 0.97	
Unemployment	Unemployed			178 (7.0%); 0.89	r = − .071***
	Employed			2367 (93.0%); 1.01	

^1^Weighting factor; ^2^r = Pearson correlation; ρ = Spearmans ρ (rho); η = eta (analysis of variance); **p < .01; ***p < .001, ^n.s^not significant

**Table 2:** Descriptive values and results of the scale analysis for the values of the SF-8 single items or the scale values

Scale/items	MD^1^ n|%	Number of response categories	M	SD	Skewness^2^	%Extreme^3^	r_iγ_^4^	r_itc_^5^ 1-DIM	r_itc_^5^ 2-DIM^MOD^	r_itc_^5^ 3-DIM
Physical Health (PHS)								α = .923	α_PHS2_ = .898	α_PHS3_ = .892
PF—Physical Functioning	10|0.4	5	81.20	23.81	−1.02	50.5	.134	.784	.806	.813
PR—Physical Role Functioning	9|0.4	5	85.15	22.29	−1.41	59.7	.127	.803	.797	.828
BP—Bodily Pain	9|0.4	6	80.16	23.80	−1.00	45.5	.120	.724	.741	.724

Overall Health (OHS)										α_OHS_ = .779
GH—General Heath Perception	6|0.2	6	66.92	19.63	−0.16	11.8	.169	.709	.733	.638
VT—Vitality	10|0.4	5	68.67	21.79	−0.44	17.4	.116	.680	.661	.638

Mental Health (MHS)									α_MHS_ = .854	
SF—Social Functioning	10|0.4	5	85.86	22.01	−1.38	63.1	.078	.709	.723	
MH—Mental Well-Being	12|0.5	5	86.76	21.61	−1.68	63.3	.098	.685	.749	
RE—Emotional Role Functioning	33|1.3	5	87.61	21.58	−1.81	67.5	.038^n.s^	.727	.703	

^1^Number of missing data; ^2^standard error = .049; ^3^percentage of responses in the response category that does not indicate a health problem; ^4^correlation Item × weighting factor γ; ^5^corrected item-total correlation; n.s.: not significant; all other correlations p > .001

All SF-8 items have been answered almost completely (maximum missing data rate: 1.3%). To avoid biases caused by missing data the very few missing responses were imputed by the EM algorithm [37].

### *SF-8 structure according to Ware *et al*. *[8]

Table 3 shows the results for the model specification according to the original model proposed by Ware et al. ([8]; see Fig. 1). All global fit-measures identify the *Full Model* (assigning each item to both factors) as better fitting than the *Restricted Model.* As in the original study applying PCA [8], the items *Physical Functioning*, *Physical Role Functioning*, and *Bodily Pain* were most strongly associated with the *Physical Health* component. The items *Social Functioning*, *Mental Health* and *Emotional Role Functioning* reflected the construct *Mental Health* most distinctly. In accordance with the results reported by Ware et al. [8], the items *General Health Perception* and *Vitality* showed a clear double loading in the present study. However, both items were more strongly associated with the *Mental Health* factor in the *full model*. Note, that the variance of the items *General Health Perception* (R^2^ = 0.370–0.470) and *Vitality *(R^2^ = 0.462–0.590) was explained most weakly for both model definitions.

**Table 3:** Factor loadings and global fit measures for the for the ML- and WLSMV estimates of the Full 2-DIM WIM-model and the Restricted 2-DIM WIM-model according to Ware et al. [8]

Items	Full 2-DIM WIM-model						Restricted 2-DIM WIM-model					
	ML			WLSMV			ML			WLSMV		
	PH_full_	MH_full_	R^2^	PH_full_	MH_full_	R^2^	PH_res_	MH_res_	R^2^	PH_res_	MH_res_	R^2^
PF	.753	.482	.799	.749	.560	.874	.886	–	.785	.926	–	.857
PR	.743^1^	.525	.827	.749^1^	.600	.921	.910^1^	–	.827	.962^1^	–	.925
BP	.593	.496	.593	.593	.569	.676	.780	–	.608	.833	–	.695
GH	.382	.479	.376	.395	.560	.470	.370	.278	.377	.400	.315	.469
VT	.409	.543	.462	.412	.649	.590	.371	.350	.465	.367	.435	.589
SF	.256	.749	.627	.266	.816	.737	–	.802	.644	–	.868	.754
MH	.104	.870^1^	.768	.130	.915^1^	.853	–	.808^1^	.653	–	.861^1^	.741
RE	.334	.723	.634	.351	.800	.764	–	.819	.671	–	.906	.821
r_PH,MH_	.000			.000			.789			.839		
χ^2^	215.52			263.66			363.26			416.67		
df	12			12			17			17		
TLI	.962			.995			.954			.986		
CFI	.984			.988			.972			.992		
RMSEA (90%-CI)	[.072; .082; .091]			[.080; .089; .098]			[.082; .089; .098]			[.088; .096; .104]		
SRMR	.022			.013			.028			.017		
BIC	43,804.39			–			44,014.59			43,809.03		

^1^Unstandardized coefficients restricted (value = 1) to ensure identifiability; *PH* Physical Health, *MH* Mental Health, *OH* Overall Health, *r*_*PH,MH*_ latent correlaton of the factors PH and MH

### Confirmatory factorial analyzes of the SF-8 structure

Table 4 shows the results for the CFA model structures assuming BIM. For both estimation methods, similar differences in the model quality criteria were found. In the following, we refer primarily to the WLSMV-estimates which are based on more valid distributional assumptions. In accordance with study results reported by Lang et al. [10] the best data fit was found for the three-dimensional CFA model (χ^2^_df=19_ = 248.68; CFI = 0.995; RMSEA = 0.073; SRMR = 0.014). The modified two-dimensional model (2-DIM^MOD^; χ^2^_df=19_ = 622.50; CFI = 0.987; RMSEA = 0.112; SRMR = 0.024) assuming *Vitality* to be an indicator of *Physical Health* allowed for a better data fit, than the two-dimensional BIM-model (2-DIM; χ^2^_df_ = 19 = 730.83; CFI = 0.985; RMSEA = 0.112; SRMR = 0.026).

**Table 4:** Factor loadings and measures of global fit for the ML- and WLSMV-estimates of the models assuming between-item-multidimensionality (BIM); 1-, 2-, 3-DIM = one-, two- and three-dimensional model specification

	1-DIM		2-DIM				2-DIM^MOD^				3-DIM					
	ML	WLSMV	ML		WLSMV		ML		WLSMV		ML			WLSMV		
Items			PH	MH	PH	MH	PH	MH	PH	MH	PH	OH	MH	PH	OH	MH
PF	.849^1^	.912^1^	.882	–	.912	–	.878	–	.919	–	.886^1^	–	–	.957^1^	–	–
PR	.875	.942	.910^1^	–	.942^1^	–	.899^1^	–	.953^1^	–	.913	–	–	.962	–	–
BP	.773	.808	.778	–	.808	–	.783	–	.821	–	.778	–	–	.835	–	–
GH	.624	.679	.600	–	.679	–	.614	–	.691	–	–	.690^1^	–	–	.730^1^	–
VT	.689	.759	–	.676	–	.789	.675	–	.776	–	–	.778^1^	–	–	.821^1^	–
SF	.718	.825	–	.794	–	.853	–	.805	–	.870	–	–	.804^1^	–	–	.870^1^
MH	.690	.831	–	.790^1^	–	.851^1^	–	.806^1^	–	.864^1^	–	–	.809	–	–	.863
RE	.757	.855	–	.814	–	.889	–	.822	–	.908	–	–	.820	–	–	.980
r_PH, MH_			.812		.853		.836		.872		.821			.866		
r_PH,OH |_ r_MH,OG_											.889 | .843			.906 | .891		
χ^2^	1312.99	1303.82	582.69		730.83		509.44		622.15		254.97			248.68		
df	20	20	19		19		19		19		17			17		
TLI	.855	.962	.934		.978		.942		.981		.981			.992		
CFI	.897	.973	.955		.985		.961		.987		.969			.995		
RMSEA (90%-CI)	[.152; .155; .167]	[.152; .159; .166]	[.101; .108; .116]		[.114; .122; .129]		[.093; .101; .108]		[.104; .112; .119]		[.066; .074; .082]			[.065; .073; .081]		
SRMR	.050	.038	.045		.026		.035		.024		.022			.014		
BIC	44,940.79	–	44,218.33		–		44,145.09		–		43,906.30			–		

^1^Unstandardized coefficients restricted (value = 1) to ensure identifiability; *PH* Physical Health, *MH* Mental Health, *OH* Overall Health, *r*_*x,y*_ latent correlation of the factors X and Y

### Bi-factor models of the SF-8 structure

For the bi-factor models, both the two-dimensional modified model (2-DIM^MOD^) and the three-dimensional model (3-DIM) show a considerably better data fit than BIM models. In particular, the χ^2^-values (71.073_df = 12_, 92.38_df = 13_), the RMSEA (0.044, 0.049) value and the SRMR (0.001) are significantly lower than for the BIM models (Table 4). The BIC which can only be determined for the ML-estimation also identified the bi-factor models as best fitting.

In the bi-factor models, all SF-8 items are associated with the general factor (loadings: 0.698–0.873) to a much higher degree than with the specific factors *Physical*, *Mental* and, if applicable, *Overall Health* (loadings: 0.216–0.582). The general factor, which can be interpreted in terms of the general HRQoL, thus proves to be the dominant source of the item variances (Table 5).

**Table 5:** Factor loadings and global fit measures for the for the ML- and WLSMV estimates of the bifactor-models assuming within-item-multidimensionality (WIM; all factor assumed to be uncorrelated)

Items	Bi-factor 2-DIM^MOD^								Bi-factor 3-DIM									
	ML				WLSMV				ML					WLSMV				
	GEN	PH	MH	R^2^	GEN	PH	MH	R^2^	GEN	PH	OH	MH	R^2^	GEN	PH	OH	MH	R^2^
PF	.841^1^	.212	–	.752	.873^1^	.289	–	.846	.792^1^	.405	–	–	.791	.841^1^	.393	–	–	.861
PR	.881	.359^1^	–	.905	.908	.371^1^	–	.963	.821	.411^1^	–	–	.843	.873	.423^1^	–	–	.942
BP	.772	.068	–	.601	.808	.155	–	.653	.764	.216	–	–	.604	.790	.234	–	–	.678
GH	.658	−.190	–	.470	.710	−.093	–	.513	.639	–	.255^1^	–	.474	.698	–	.224^1^	–	.538
VT	.742	−.257	–	.616	.821	−.206	–	.716	.713	–	.297^1^	–	.596	.786	–	.224^1^	–	.668
SF	.669	–	.428	.631	.762	–	.401	.741	.701	–	–	.367	.626	.789	–	–	.337^1^	.736
MH	.631	–	.608^1^	.768	.735	–	.566^1^	.861	.668	–	–	.582^1^	.786	.761	–	–	.555	.887
RE	.715	–	.351	.635	.814	–	.401	.767	.749	–	–	.282	.641	.843	–	–	.251	.774
χ^2^	81.90				71.07				126.34					92.38				
df	12				12				13					13				
TLI	.987				.997				.981					.996				
CFI	.994				.999				.991					.998				
RMSEA (90%-CI)	[.038; .048; .058]				[.034; .044; .054]				[.049; .059; .068]					[.040; .049; .059]				
SRMR	.011				.001				.013					.001				
BIC	43,772.44				–				43,809.03					–				

^1^Unstandardized coefficients restricted (value = 1) to ensure identifiability; *PH* Physical Health, *MH* Mental Health, *OH* Overall Health

### Calculation of the SF-8-scale scores

According to these results, five scale scores (T-values: M = 50; SD = 10) can be calculated representing the information of the SF-8 items according to the 2-DIM^MOD^ model and the 3-DIM model (BIM models) as well as the bi-factor specification. The syntax for calculating these scale scores is attached in the Additional file 1. The *Mental Health Score (MHS)* (*α* = 0.854) aggregates the item group identified as homogeneous in both the 2-DIM^MOD^ and 3-DIM models: *Social Functioning*, *Mental Health* and *Emotional Role Functioning*.

The *Physical Health Score* (PHS2) (*α* = 0.898) represents the information of the items *Physical Functioning*, *Physical Role Functioning*, *Bodily Pain*, *General Health Perception* and *Vitality* according to the 2-DIM^MOD^ model. According to the 3-DIM model, the *Physical Health Score* (PHS3) (*α* = 0.892) aggregates the information of the items *Physical Functioning*, *Physical Role Functioning* and *Bodily Pain*. *Overall Health* (*α* = 0.779) represents *General Health Perception* and *Vitality*. The *SF-8 total score* (*α* = 0.918) combines the information of all 8 items to a general indicator of HRQoL. Table 2 shows the item-total correlation for each scale definition.

Table 6 displays the correlation of these scale scores and the scale scores *Physical Component Summary* (*PCS)* and *Mental Component Summary (MCS)* according to Ware et al. [8]. As expected *PCS* was very strongly associated with the physical scores *PHS2* (*r* = 0.960) and *PHS3* (*r* = 0.973), respectively. *MCS* values corresponded very highly with the mental score *MHS* (*r* = 0.939). *OHS* and *SF-8-Total* were more strongly correlated with *PCS* than with *MCS*. Generally, all scale scores were highly intercorrelated (*r* ≥ 0.633), which underlines the high commonality of the HRQoL-related information collected by the SF-8 items.

**Table 6:** Correlation of the SF-8 scale scores PCS and MCS proposed by Ware et al. [8] and the scale scores based on the Bi-factor models

	M	SD	Skewness^1^	PHS (2-DIM)	PHS (3-DIM)	OHS	MHS	SF-8 total
PCS	50.31	8.40	−1.331	.960	.974	.771	.599	.882
MCS	53.25	7.83	−1.731	.613	.513	.647	.939	.794
PHS (2-DIM)	50.00	10.00	−0.943	*.898*^2^	.954	.893	.716	.957
PHS (3-DIM)	50.00	10.00	−1.497		*.892*	.718	.689	.918
OHS (3-DIM)	50.00	10.00	−1.121			*.779*	.633	.852
MHS	50.00	10.00	−1.168				*.854*	.887
SF-8 Total	50.00	10.00	−1.211					*.918*

^1^Standard error of skewness = .049; ^2^Cronbachs α in the diagonal (italics)

## Discussion

In this study, a satisfactory fit of the SF-8 to different model specifications could be confirmed by means of CFA in a German general population sample. Ware et al. ([8]; see Fig. 1) suggest that the SF-8 data can be summarized as a Physical and a Mental health component score. The according Full 2-DIM WIM-model assuming double loadings for all 8 items (Table 3) exhibited a slightly better model-fit than the restricted model definition. The explained proportion of variance is weakest for the items *Vitality* and *General Health Perception* (0.469–0.590).

Alternatively, models assuming each item to be indicative for only one of the underlying latent factors (BIM) also showed a good data fit (Fig. 2, Table 4). Assuming BIM, the best CFA model fit has been identified for the three-factor model structure (3-DIM) reported by Lang et al. [10]. The third factor, *Overall Health*, is formed by the two items *General Health Perception* and *Vitality*.

For the two-dimensional BIM definition, the assignment of the item *Vitality* to the physical factor in the model 2-DIM-Modified lead to an improved data fit. This is in accordance with the results of Lang et al. [10] in a representative Chinese population. Lang et al. [10]) discuss these results as particularly characteristic for the Asian region (see also: [9, 11–13, 15]) in comparison to European and US-American data. The findings reported in the present paper provide evidence that cultural differences should not be assumed as the main cause for differences in the reported findings. The well-founded CFA approach of Lang et al. [10] yields very similar results in the Chinese population as the CFA approach in the data presented here for Germany. Differences to earlier analyses in the United States [8, 38], thus seem to be due to the CFA approach.

For the short versions SF-12 and SF-8, high correlations of the *Physical*)PCS) and *Mental Component Summary* (MCS) are reported in the literature [8–10, 19]. Despite this high correlation of *Physical Health* and *Mental Health*, a general factor HRQoL has not yet been considered when evaluating the SF-8. The underlying assumption of the BIM is: Each item exclusively covers either a *physical* or *mental* aspect. If the bi-factor approach (WIM assumption) is applied, a fundamentally different model is assumed. Bi-factor models allow the information of the SF-8 items to be determined by general HRQoL. Our findings showed a clearly better data fit for the bi-factor models (Table 5). Note, that in these models *Physical* and *Mental* as well as *Overall Health* are assumed to be uncorrelated components. The correlation of the single items assigned to different facets is completely modeled by the general factor HRQoL. In the bi-factor models our results showed, that the general HRQoL dominantly determines the variance of all items. WIM thus represents a plausible and statistically superior model assumption, which opens a completely new view on the structure of the SF-8 [22, 23, 25]: The SF-8 primarily measures a general HRQoL component. Assuming a dominant principal component HRQoL for the items of SF-8 is further supported by the results of a PCA: Only the eigenvalue 5.11 of the first component is greater than 1. This first component explains a very high amount of the item variances: 63.40%.

Accordingly, a SF-8 overall score can be determined, which represents HRQoL across physical and mental facets. This approach thus represents a psychometrically well-founded alternative to existing evaluation approaches for scale variants of the SF family. The suitability should also be tested for the SF-12 and SF-36.

At the level of global model fit measures, the two-factor model (Bi-factor 2-DIM^MOD^) allows for a better data fit than the three-factor model (Bi-factor 3-DIM) (Table 4). However, this superiority is not supported by the item loading structure. In contrast to the items *Physical Functioning*, *Physical Role Functioning* and *Bodily Pain* (loadings: 0.289, 0.371, 0.155), the two items *General Health Perception* and *Vitality* were associated negatively (loadings: −0.093, −0.206) with the factor *Physical Health*. Accordingly, these two items proved to be indicators of the general health factor rather than specific health factors.

The model estimates were calculated using both the ML algorithm as well as the WLSMV algorithm. Generally, the global fit measures (especially χ^2^, CFI and SRMR) indicated a better model fit for the WLSMV estimates. The poorer fit for the ML estimates was expected because of strong violations of the normal distribution in the analyzed norm data set. The WLSMV algorithm is methodologically superior to alternative modeling approaches when the underlying latent correlation structure is analyzed. WLSMW prevents underestimation of correlations due to asymmetric data distributions and categorical data format [30, 33, 34]. Accordingly, applying the WLSMV algorithm leads to factor loadings and explained item variances being higher. The validity of all modeling results is systematically attenuated when the ML approach is used in the case of clearly non-normal distributed data [27, 28].

Some limitations of the study must be considered in order to correctly assess the study results. We focused on the dimensional structure of the SF-8, without analyzing further clinimetric characteristics of the instrument [39]. Clinimetrics emphasizes, that each assessment has to be evaluated regarding its suitability for specific purposes in clinical practice comprehensively. In addition to our study results, it would be particularly important to find out to what extent the individual items as well as the scale scores of the SF-8 are able to reflect clinically relevant changes in health status validly over time. In addition, future research should focus on how the SF-8 can be embedded in an overall assessment to address individual patient needs in treatment planning and to sensitively evaluate clinically significant changes [40, 41].

## Conclusions

For the SF-8, the fit to two-factorial (*Physical* and *Mental Health*) and three-factorial latent structure models (in addition: *Overall Health*) could be substantiated in a German general population sample. Furthermore, a good model fit was achieved using bi-factor models, in which the generic construct *HRQoL* is shown to be a major source of variance in each of the SF-8 items. Accordingly, the *SF-8 Total Score* may be a valid way of summarizing the SF-8 data indicating general *HRQoL*. Future studies should evaluate the usefulness of the *SF-8 Total Score* in quantifying disease burden and evaluating clinically significant changes.

## Supplementary Information

**Additional file 1**. SF8_scoring_syntax.doc; SPSS syntax file for calculation the SF-8 scale scores.

## Data Availability

The data sets used and analyzed during the current study are available from the corresponding author on reasonable request.

## Abbreviations

BIC: Bayesian information criterion
BIM: Between-item-multidimensionality
BP: Bodily Pain
CFA: Confirmatory factor analysis
CFI: Comparative-fit-index
DF: Degrees of freedom
DIM: Dimension
EM: Expectation–maximization
GH: General Health
HRQol: Health-related quality of life
MCS: Mental Component Summary
MH: Mental Health
MHS: Mental Health Score
ML: Maximum-likelihood
MLR: Restricted maximum-likelihood
OHS: Overall Health Score
PCA: Principal components analysis
PCS: Physical component summary
PHS: Physical Health Score
PF: Physical Functioning
QoL: Quality of life
RE: Emotional Role Functioning
RP: Physical Role Functioning
RMSEA: Root mean square error of approximation
SF: Social Functioning
SF-8, SF12, SF-36: Short Form Survey
SRMR: Standardizes root mean residual
TLI: Tucker–Lewis-Index
VT: Vitality
WIM: Within-item-multidimensionality
WLSMV: Weighted-least-square-means-and-variances
