# Supplementary material for: Confirmatory and bi-factor analysis of the Short Form Health Survey 8 (SF-8) scale structure in a German general population sample
Source: Health Qual Life Outcomes. 2021 Mar 3;19:73. doi: 10.1186/s12955-021-01699-8 (PMC7931558; doi:10.1186/s12955-021-01699-8)
Supplement: Supplementary file 1 — Additional file 1. SF8_scoring_syntax.doc; SPSS syntax file for calculation the SF-8 scale scores. [file 12955_2021_1699_MOESM1_ESM.docx]

**Additional file: SPSS Scoring Syntax**

Original data according to the coding in Tab. 2. 0 = minimum healthy; 100 = maximal healthy

* For all items high values indicate high health-related quality of life.

* z Standardization of the 8 individual indicators on which the scale value formation is based. (Mean = 0, SD =1)

COMPUTE GH_z=(GH-66.9179)/19.63123.

COMPUTE PF_z=(PF-81.2011)/23.80984.

COMPUTE PR_z=(PR-85.1481)/22.29416.

COMPUTE BP_z=(BP-80.1561)/23.79632.

COMPUTE VI_z=(VI-68.6695)/21.78980.

COMPUTE SF_z=(SF-85.8591)/22.01001.

COMPUTE MW_z=(MW-86.7536)/21.61283.

COMPUTE ER_z=(ER-87.6149)/21.58457.

*Calculation of the standardized scale values (Mean = 0)

COMPUTE PHS_z_cfa2 = mean(GH_z, PF_z, PR_z, BP_z, VI_z).

COMPUTE PHS_z_cfa3 = mean(PF_z, PR_z, BP_z).

COMPUTE OHS_z_cfa3 = mean(GH_z, VI_z).

COMPUTE MHS_z_cfa23 = mean(SF_Z, MH_z, ER_z).

COMPUTE SF8_total_z = mean(GH_z, PF_z, PR_z, BP_z, VI_z, SF_Z, MW_z, ER_z).

*Calculation of the T-values of the scales (Mean = 50; SD = 10)

COMPUTE PHS2_T = ((10/0.84247) * PHS_z_cfa2) + 50.

COMPUTE PHS3_T = ((10/0.90654) * PHS_z_cfa3) + 50.

COMPUTE OHS3_T = ((10/0.90503) * OHS_z_cfa3) + 50.

COMPUTE MHS_T = ((10/0.87946) * MHS_z_cfa) + 50.

COMPUTE SF8_T = ((10/0.79677) * SF8_z) + 50.

VAR LAB PHS2_T 'Scale Physical Health; 2-DIM)‘.

VAR LAB PHS3_T 'Scale Physical Health; 3-DIM)‘.

VAR LAB OHS2_T 'Scale Overall Health)‘.

VAR LAB MHS_T 'Scale Mental Health)‘.

VAR LAB SF8_T 'SF8 Total Score‘.

EXECUTE.
